# 

**Published:** 1810-02

**Authors:** 


### CORREPONDENCE.

We are obliged to T. for his ingenious communication, and agree with him that an anonymous answer to a real correspondent may be sometimes admissable. We think, however, on further reflection, T. will see that his remarks might be more gentle. Perhaps he will put them in the form of queries, which will have the double advantage of leading the gentleman he addresses to reconsider his case, and also to feel flattered by being referred to for further information. We are indeed so much pleased with T's paper that we should gladly insert all his remarks in the form of an essay, without any, or with only a side referrence to the Correspondent he wishes to set right.

